# Effects of Mesenchymal Stem Cell-Derived Exosomes on Experimental Autoimmune Uveitis

**DOI:** 10.1038/s41598-017-04559-y

**Published:** 2017-06-28

**Authors:** Lingling Bai, Hui Shao, Hongxing Wang, Zhihui Zhang, Chang Su, Lijie Dong, Bo Yu, Xiteng Chen, Xiaorong Li, Xiaomin Zhang

**Affiliations:** 1 Tianjin Medical University Eye Hospital, Eye Institute &School of Optometry and Ophthalmology, Tianjin, 300384 P.R. China; 2 Department of Ophthalmology and Visual Sciences, Kentucky Lions Eye Center, University of Louisville, Louisville, KY 40202 USA; 3 Department of Ophthalmology, Chuiyangliu Hospital, Beijing, China

## Abstract

We previously demonstrated that mesenchymal stem cells (MSCs) ameliorated experimental autoimmune uveoretinitis (EAU) in rats. Recently, MSC-derived exosomes (MSC-Exo) were thought to carry functions of MSCs. In this study, we tested the effect of local administration of human MSC-Exo on established EAU in the same species. Rats with EAU induced by immunization with interphotoreceptor retinol-binding protein 1177–1191 peptide were treated by periocular injections of increasing doses of MSC-Exo starting at the disease onset for 7 consecutive days. The *in vitro* effects of MSC-Exo on immune cell migration and responder T cell proliferation were examined by chemotactic assays and lymphocyte proliferation assays, respectively. We found that MSC-Exo greatly reduced the intensity of ongoing EAU as their parent cells by reducing the infiltration of T cell subsets, and other inflammatory cells, in the eyes. Furthermore, the chemoattractive effects of CCL2 and CCL21 on inflammatory cells were inhibited by MSC-Exo. However, no inhibitory effect of MSC-Exo on IRBP-specific T cell proliferation was observed. These results suggest that MSC-Exo effectively ameliorate EAU by inhibiting the migration of inflammatory cells, indicating a potential novel therapy of MSC-Exo for uveitis.

## Introduction

Mesenchymal stem cell (MSC)-based therapy for the treatment of autoimmune diseases has demonstrated a particular promise after its successful applications in both animal models and patients. Accumulative evidence has established that the secretion of inhibitory soluble factors is the principle mechanism of MSC immunosuppressive activity^1, 2^. Our group recently reported that CD73 on the cell membrane also contributes to the immunomodulatory capacity of MSCs^3^, suggesting that MSCs exert their immunomodulatory properties in a multifactorial manner.

The finding that culture medium collected from MSCs could confer therapeutic effects in animal models generated on-going interests in the analysis of the components of the culture medium^4, 5^. It was recently revealed that exosomes derived from MSCs (MSC-Exo) play important roles in mediating the biological functions of MSCs^6–9^. Exosomes are 40–100 nm soluble microvesicles with a bi-lipid membrane and a cargo abundant in proteins and RNAs, which are now believed to be important for intercellular communication^9^. Although the mechanism remains elusive, the interest in exploring the therapeutic potential of MSC-Exo has greatly increased after the first report of MSC-Exo ameliorating myocardial ischemia/reperfusion injury in a mouse model^10^. Recent studies revealed that MSC-Exo do perform a variety of functions of MSCs, and has been successfully applied in animal models of tissue injuries^10–21^, allograft rejection including allogenic skin graft rejection^22^, and even human graft-versus-host disease (GVHD)^23^; however, it has not been tested in autoimmune diseases including uveitis.

Autoimmune uveitis is a major cause of visual disability worldwide. Clinical treatment currently involves the use of corticosteroids, other immunosuppressive drugs and newly developed biologics, the long term application of which are limited by serious systemic side effects and the local risk of cataracts and glaucoma^24, 25^. Localized therapies such as periocular/intraocular injection/implantation of non-corticosteroid drugs are therefore preferred to reduce the systemic and local toxicity of currently available treatments^26^.

It has been recently shown that intravenous injection of MSCs ameliorated experimental autoimmune uveitis (EAU) in both rats and mice^27–34^. In this study, we wanted to examine the efficacy of periocular injection of human umbilical cord-derived MSC-Exo in a rat EAU model. We found that local administration of MSC-Exo could ameliorate uveitis like their parent cells. However, instead of by inhibiting specific T cell responses, MSC-Exo exerted their immunosuppressing activities by inhibition of the migration of inflammatory cells into the eye.

## Results

### Identification of MSC and MSC-Exo

**Figure 1:**
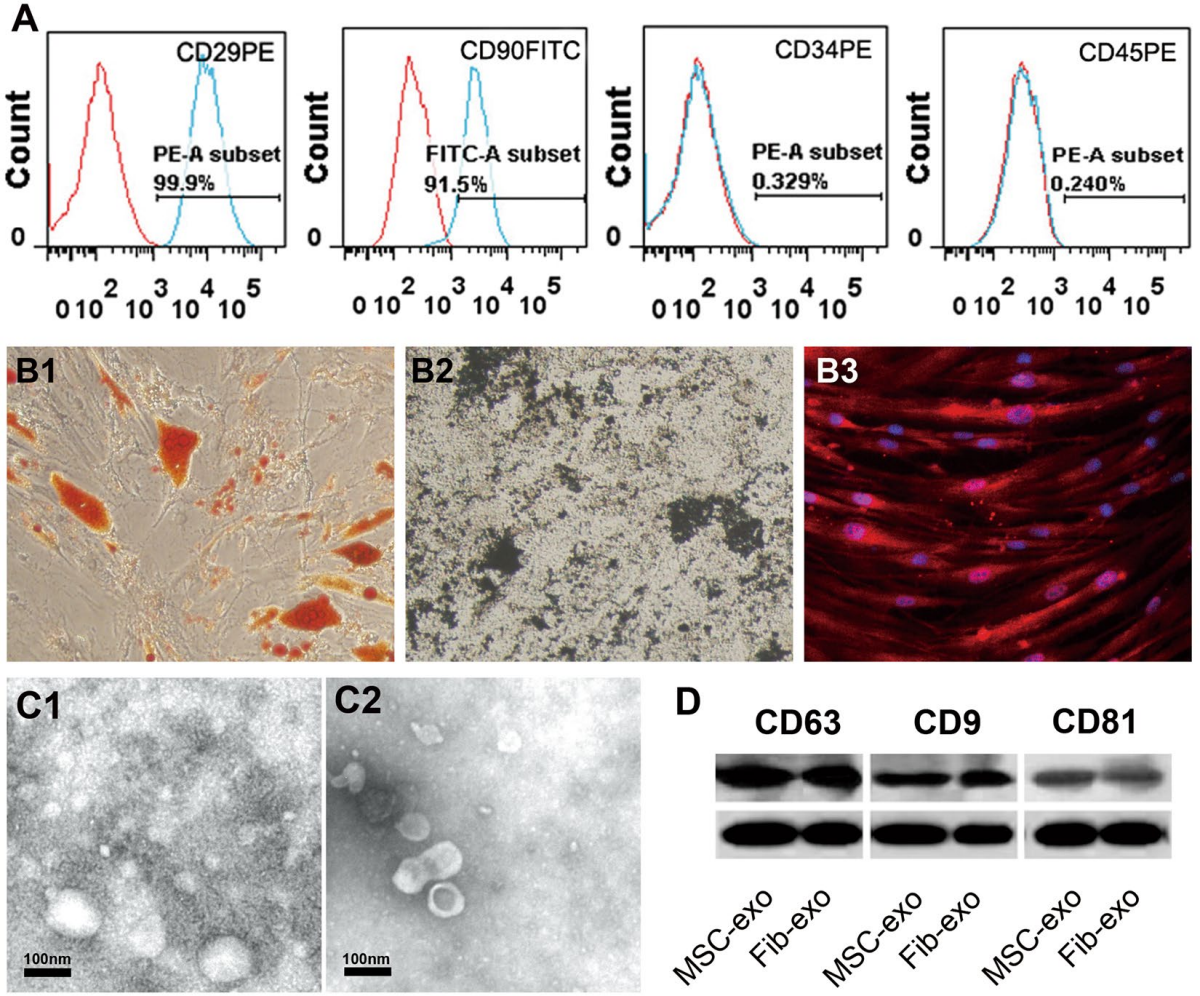
Identification of MSC and MSC-Exo. (**A**) Immunophenotypes of the cultured hMSCs were examined by flow cytometry. The vast majority of cells (>90%) were positive for CD29, CD90, but few cells (<0.5%) expressed CD34, CD45. (**B**) The hMSCs were maintained in specific induction media for 3 weeks, and the induced cells were then stained by oil red O to indicate adipocytes (B1), von Kossa for osteocytes (B2). For endothelial cell identification, the cells were firstly incubated with antibodies against vWF (B3), and then stained by secondary antibodies conjugated with Alexa Flour 647. (**C**) Micrographs of scanning electron microscopy of (C1) MSC-Exo and (C2) Fib-Exo show spheroid shaped vesicles at the diameter of about 40-100 nm. Scale bar = 200 nm. (**D**) Western blot analysis showed that both MSC-Exo and Fib-Exo express CD63, CD9 and CD81.

MSCs were analyzed by flow cytometry, which confirmed expression of CD90 and CD29, and lack of CD45 and CD34 (Fig. 1A). In addition, MSCs were functionally characterized by differentiation into adipocytes, chondrocytes, and capillary-like structures in different differentiation media (Fig. 1B). Electron microscopy demonstrated that the vesicles in our preparations of both MSCs and human dermal fibroblasts were cup-shaped and measured 40–100 nm in diameter (Fig. 1C). Western blot analysis confirmed that the vesicles from both MSCs and human dermal fibroblasts expressed markers of exosomes, CD63, CD9 and CD81.

### Periocular injection of MSC-Exo ameliorated uveitis

**Figure 2:**
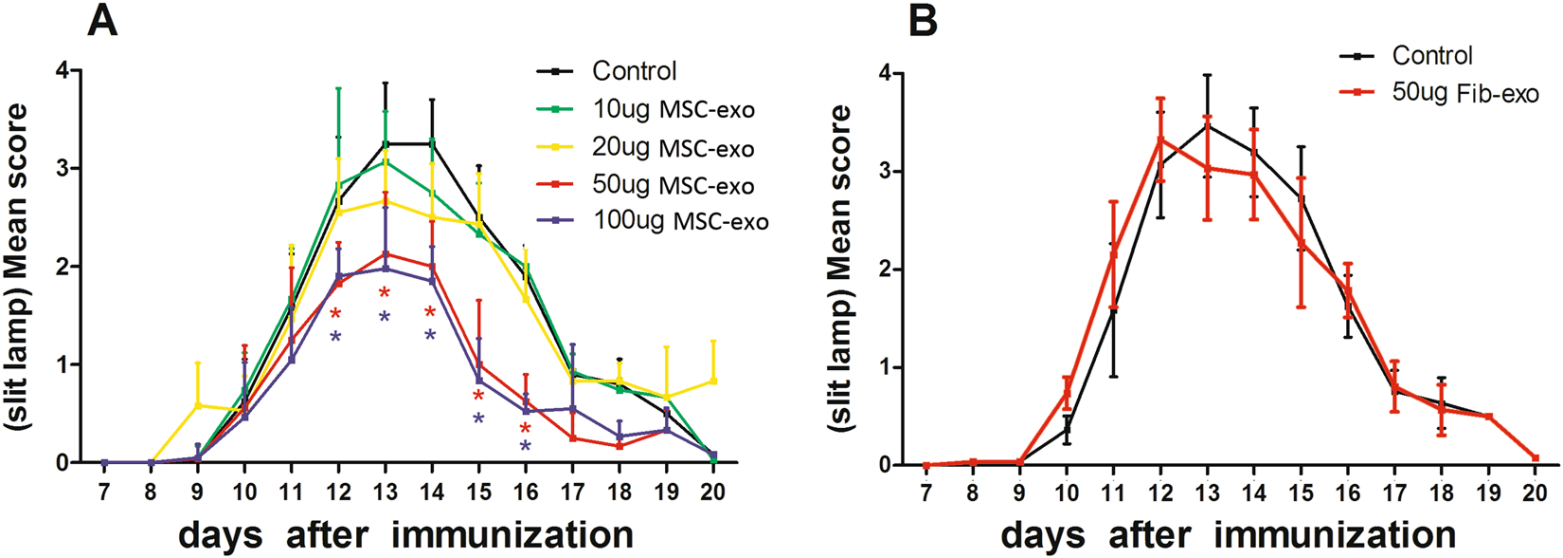
Periocular injection of MSC-Exo ameliorated uveitis in rats. (**A**) Both eyes of the immunized rats were injected periocularly with different doses of MSC-Exo (10, 20, 50, and 100 μg) or with an equal volume of PBS (control) on a daily basis for 7 consecutive days starting from the 9^th^ day post immunization. B: 50 μg FIB-exo or an equal volume of PBS was administrated in the same way as above. Asterisks indicate the significant differences between control (black curve) and exosomes treated (blue curve), Values are expressed as the mean ± SD of six rats (12 eyes) per group. *P < 0.05.

**Figure 3:**
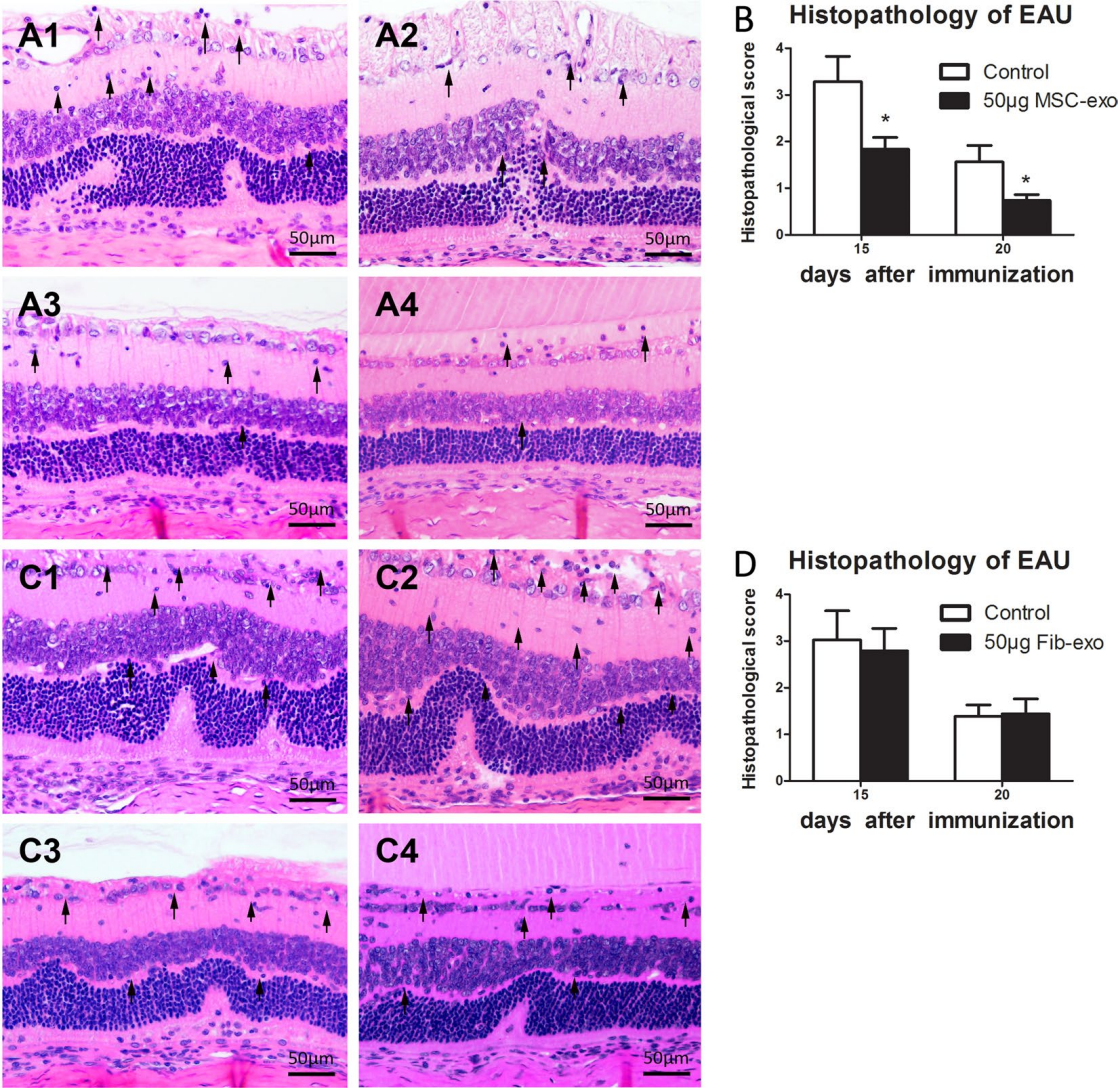
Histological assessment of the retina in EAU. Sections of the retina were stained with hematoxylin and eosin, and evaluated for histological damage on 15 and 20 days post immunization. Representative H&E staining images of PBS (A1 and C1, 15 days; B1 and B2 20 days), MSC-exo (A3, 15 days; A4, 20 days), and FIB-exo (B3, 15 days; B4, 20 days) treated groups are shown. Results are expressed quantitatively as histopathological scores (C and D). Values are expressed as the mean ± SD of six rats (12 eyes) per group. *P < 0.05.

We have previously reported that MSCs could prevent the development of EAU and suppress established EAU in rats. To examine whether exosomes released from MSCs had similar inhibitory effects on EAU as their parent cells, we periocularly injected MSC-Exo daily starting on the onset of the disease (day 9 post immunization) for 7 days. EAU rats received 50 μg Fib-Exos or an equal volume of PBS as control. As seen in the Fig. 2, while all control animals developed full disease, the rats treated with 50 or 100 μg of MSC-Exo developed significantly mild disease, as evaluated by both clinical (Fig. 2A) and pathological examinations (Fig. 3A and B). In contrast, periocular injection of Fib-Exos showed no therapeutic effects (Figs 2B and 3C,D).

**Figure 4:**
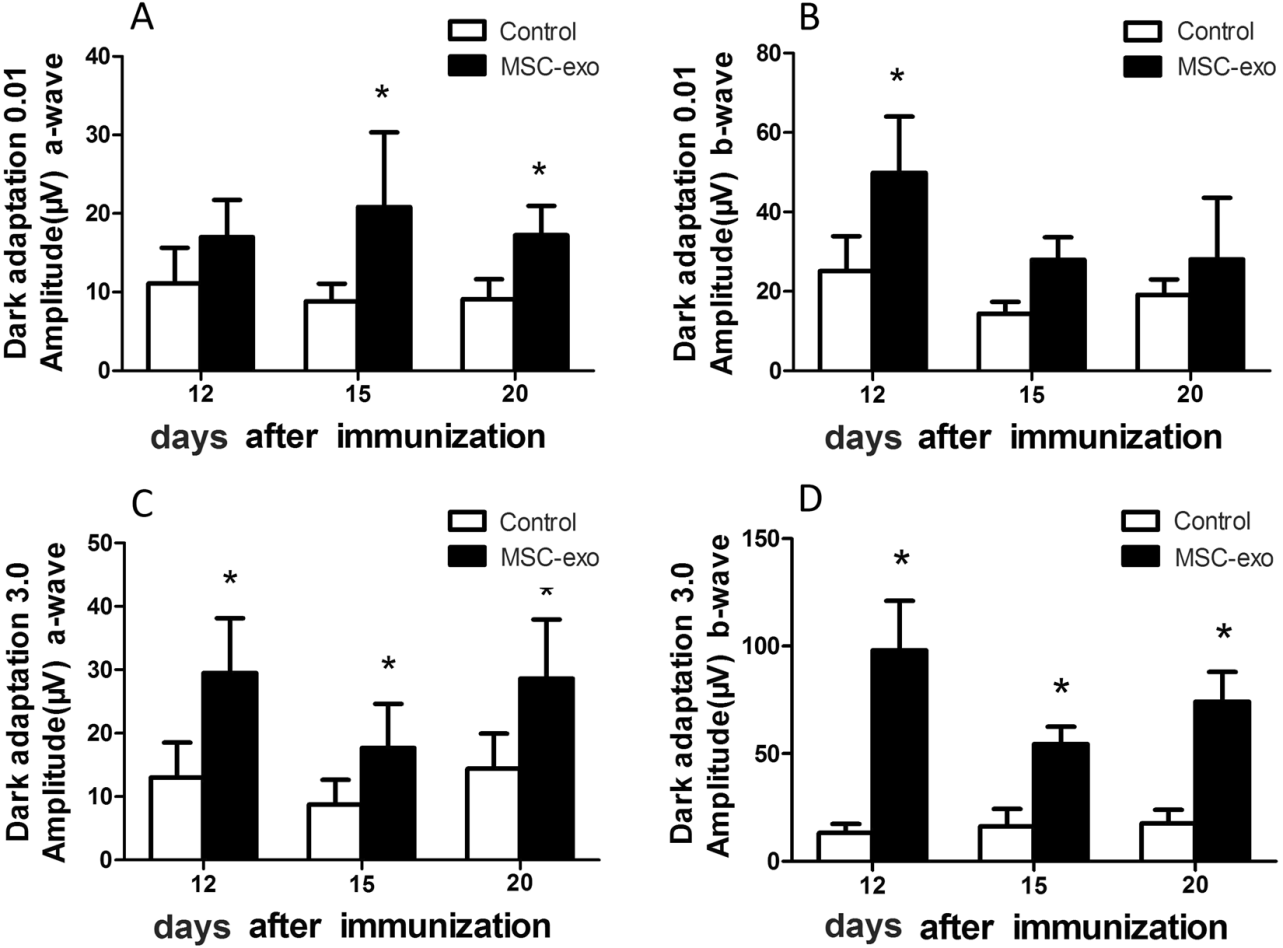
ERG records of the rats. ERG was carried out in both eyes and Ten responses to 0.01 and 3.0 cd × s/m^2^ white light flash (10 μs, 0.1 Hz) from a Ganzfeld integrating sphere were amplified and averaged. A wave (**A** and **B**) and b wave (**C** and **D**) amplitudes of dark adapted ERG at 12, 15 and 20 days post immunization were analysed and compared between MSC-exo treated group and control group. Values are expressed as the mean ± SD of six rats (12 eyes) per group. *P < 0.05.

We also performed ERG on days 12, 15, and 20 after disease induction to examine the protective role of MSC-Exo on retinal functions. As shown in Fig. 4, MSC-Exo treatment significantly improved the amplitudes of both a-waves and b-waves, especially under 3.0 intensity stimuli.

### MSC-Exo treatment reduced leukocyte infiltration in the eye

**Figure 5:**
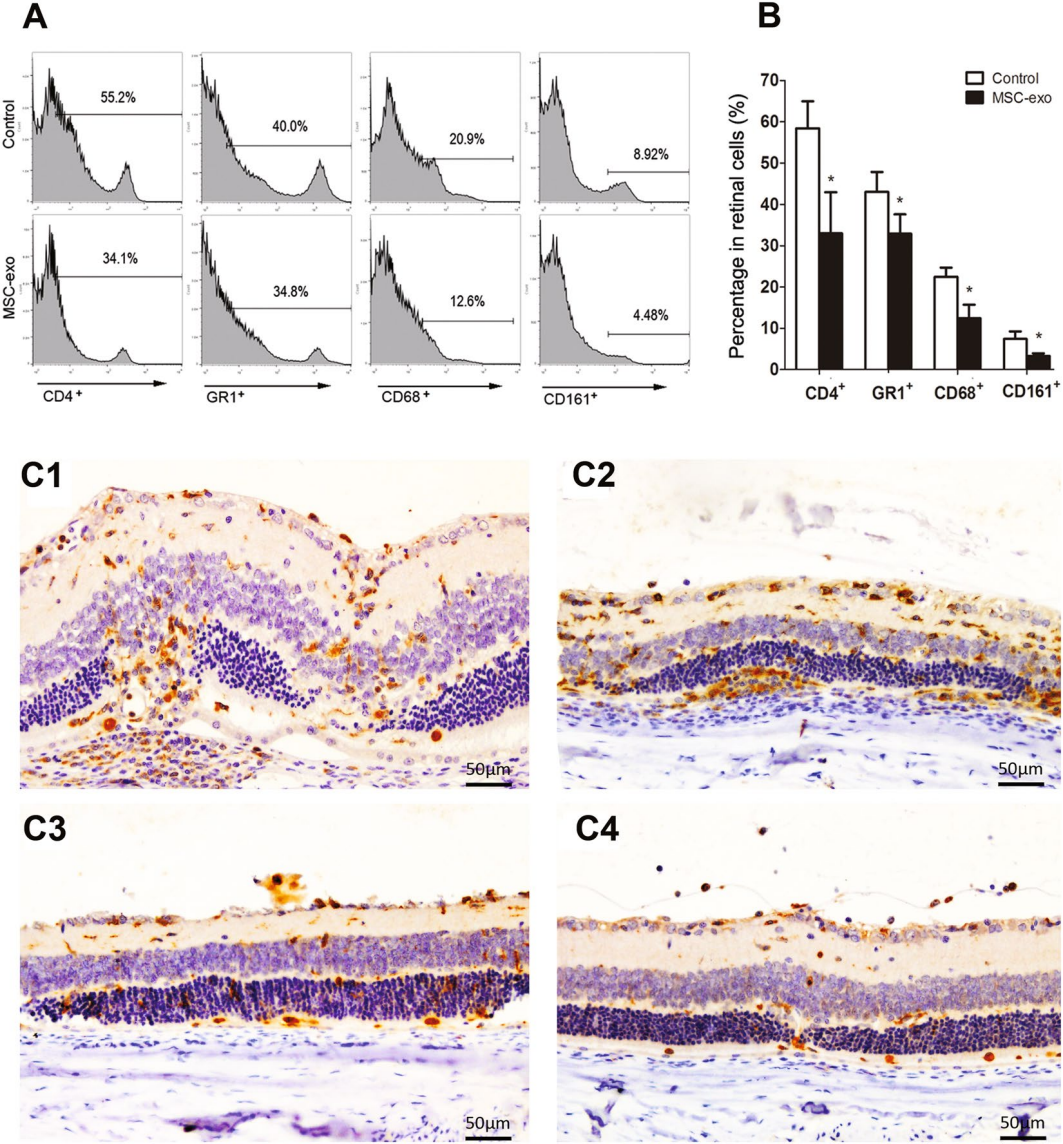
Analysis of the leukocytes infiltration in eyes. (**A** and **B**) On day 15 after immunization, representive figures of FACS analysis of CD4^+^, Gr-1^+^, CD161^+^ and CD68^+^ cells in eyes; C: CD68 immunostaining was used to observe macrophage infiltration in the eyes on day 15 and 20 after immunization (C1: control goup, day 15; C2: control group, day 20; C3: MSC-exo treated group, day 15; C4: MSC-exo treated group, day 20). Values are expressed as the mean ± SD of six rats (12 eyes) per group. *P < 0.05.

**Figure 6:**
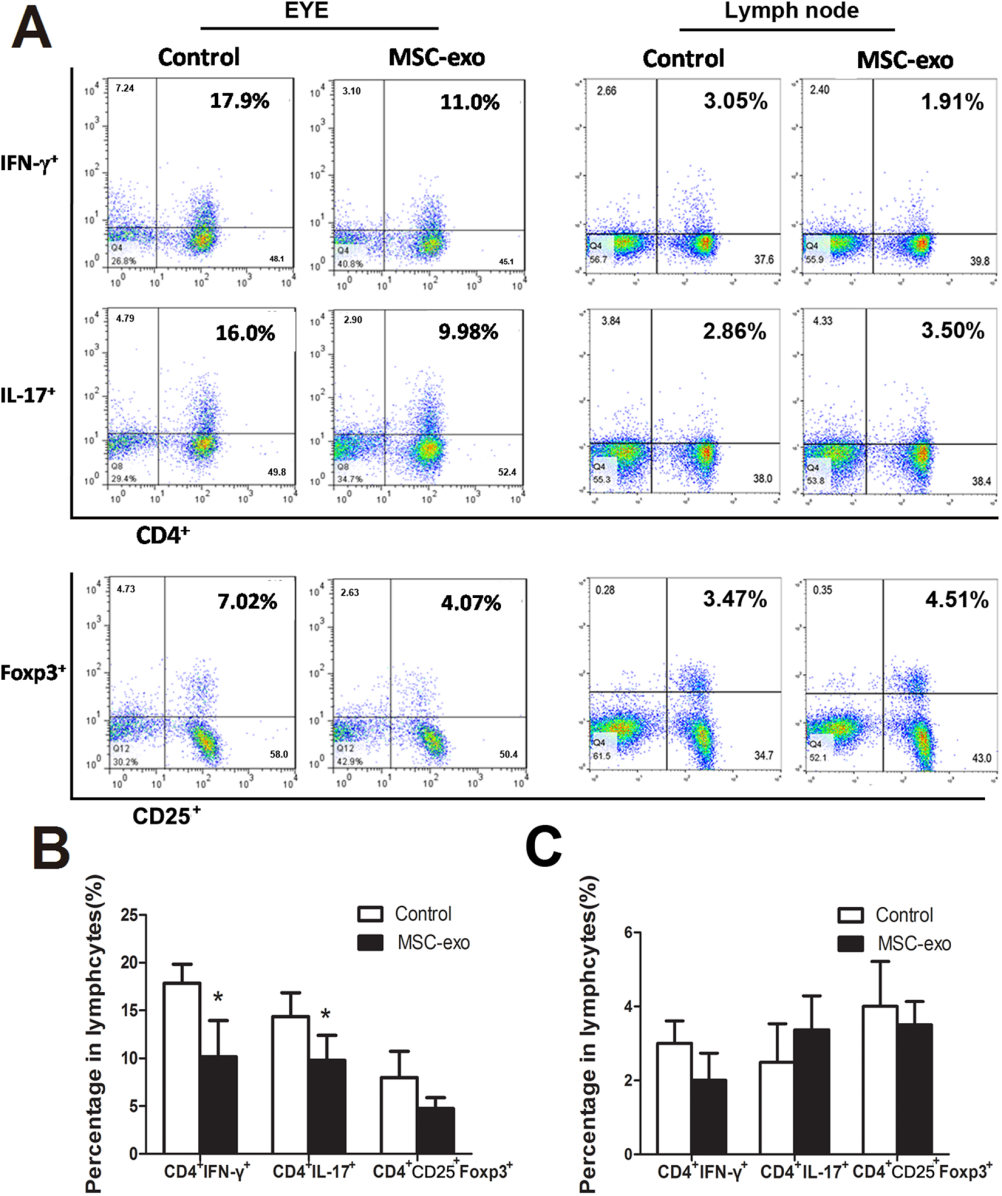
Analysis of T cell subsets from eye and lymph node. For CD4^+^CD25^+^Foxp3^+^ cell detection, cells were first gated on CD4^+^ cells, and then gated on CD25^+^ and Foxp3^+^ cells. (**A**) Representive figures of intracellular staining of IFN-γ, IL-17 and CD25^+^Foxp3^+^ in eyes-derived CD4^+^T cells (left) and in lymph nodes-dvrived CD4^+^T cells (right) from Control and MSC-exo treated rats with EAU. (**B**) the percentage of CD4^+^IFN-γ^+^, CD4^+^IL-17^+^ and CD4^+^CD25^+^Foxp3^+^ cells in eyes. (**C**) the percentage of CD4^+^IFN-γ^+^, CD4^+^IL-17^+^ and CD4^+^CD25^+^Foxp3^+^ cells in cervical draining lymph nodes. Values are expressed as the mean ± SD of six rats (12 eyes) per group. *P < 0.05.

To further determine the effects of MSC-Exo treatment on the inflammation of the retina, we assessed the frequency of CD4^+^IFN-γ^+^, CD4^+^Foxp3^+^, CD4^+^IL-17^+^, Gr-1^+^, CD161^+^ and CD68^+^ cells in eyes on day 15 after immunization, which represent Th1 cells, Treg cells, Th17 cells, granulocytes, NK cells and macrophages respectively. T cell subsets in lymph nodes including CD4^+^IFN-γ^+^, CD4^+^Foxp3^+^ and CD4^+^IL-17^+^ cells were also examined simultaneously by flow cytometry. As shown in Fig. 5, MSC-Exo treatment downregulated the proportions of Gr-1^+^, CD161^+^, CD68^+^ and CD4^+^ cells in the retina (Fig. 5A,B), Down-regulation of MSC-Exo on the macrophage migration to the retina was further verified by immunohistochemistry (Fig. 5C). MSC-Exo treatment also downregulated the proportions of CD4^+^IFN-γ^+^ and CD4^+^IL-17^+^ cells in the retina (Fig. 6A,B), while exert no effect on T cells in lymph nodes (Fig. 6A,C).

**Figure 7:**
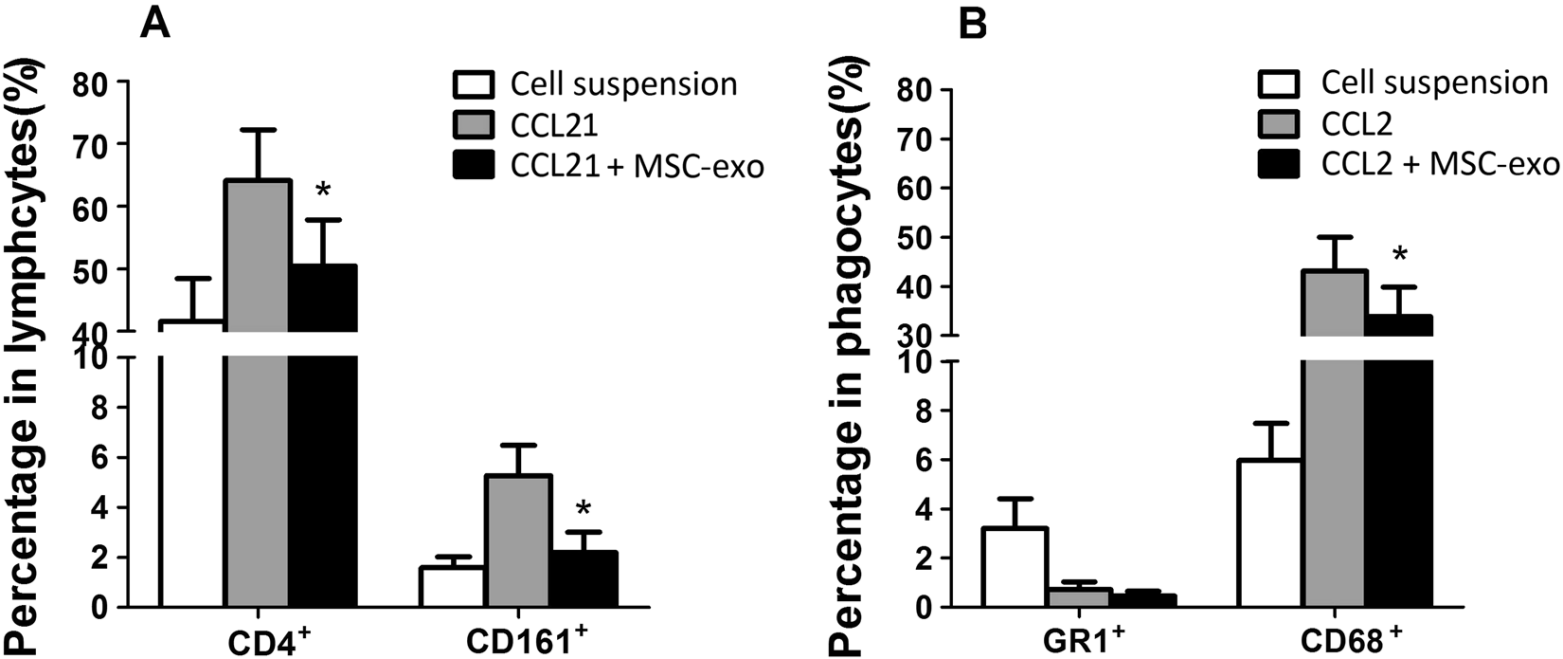
The effect of MSC-exo on the chemotaxis of leukocytes. IRBP-specific spleen monocytes prepared from immunized rat on day 12 post immunization were stimulated for 2 days with R16, and then added to the upper well of a microchemotaxis device and incubated in RPMI 1640 medium containing CCL2 or CCL21 with or without MSC-Exo (10 ug/ml). The cells that migrated to the lower well after 4 h were collected and the number of CD4^+^ (**A**), CD161^+^ (**A**), CD68^+^ (**B**) and Gr^+^ (**B**) cells were determined by flow cytometry. *P < 0.05.

We further examined the chemoattractive effect of MSC-Exo on inflammatory cells by an *in vitro* chemoattractant assay. The indicator cells were splenocytes prepared from Lewis rats immunized 12 days earlier with R16. CCL2, also called monocyte chemotactic protein-1 (MCP-1), is best known as a chemotactic agent for mononuclear cells. CCL21 (a ligand for CCR7) attracts both resting and activated T cells. As shown in Fig. 7, MSC-Exo significantly inhibited both CCL2 and CCL21 induced chemotaxis of splenocytes.

### No inhibitory effect of MSC-Exo on the proliferation of IRBP-specific T cells

**Figure 8:**
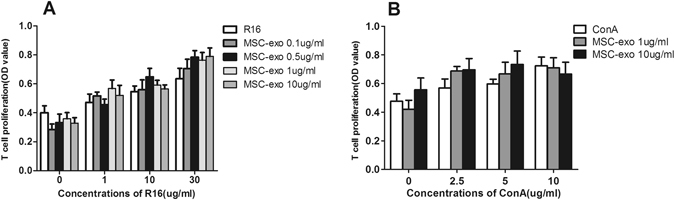
The effect of MSC-eso on T cell proliferation. T cells and APCs were prepared on day 12 after immunization, from lymph nodes and spleens of EAU rats, and were incubated (T cells/APCs, 1:1) for 48 hours with graded doses of R16 (0, 1, 10 and 30 μg/ml) and MSC-Exo (0, 0.1, 0.5, 1 and 10 μg/ml) (**A**), or with ConA (0, 2.5, 5, 10 μg/ml) and MSC-Exo (1 and 10 μg/ml). T cell proliferation was determined by measurement of BrdU incorporation. *P < 0.05.

MSCs have been reported to inhibit the proliferation of activated lymphocytes. To determine whether MSC-Exo have similar inhibitory effect on T cells as their parent cells, we examined the proliferation of splenic T cells from rats with EAU in response to increasing doses of R16 or ConA. Our data in Fig. 8 showed no inhibition of MSC-Exo, at any concentrations, on T cell proliferation challenged with either specificR16 or non-specific ConA stimulation.

## Discussion

In this study we have shown, for the first time, that local application of MSC-Exo can inhibit the autoimmune response *in vivo* using a rat model of EAU. We found that periocular injection of human MSC-Exo can reduce leucocyte infiltration, protect retinal structure and rescue retinal function of rats with EAU. The results of these studies indicate that MSC-Exo may be useful for treating human autoimmune uveitis, for which local non-corticosteroid therapy is urgently needed. Importantly, xenogeneic MSC-Exo from humans was effective in ameliorating autoimmune response in rats.

The immunosuppressive effect of exosomes has been confirmed *in vivo* in mouse allogeneic skin grafting models, in which subcutaneous injection of exosomes isolated from human embryonic stem cell-derived MSCs delayed the occurrence of GVHD for 2 days, concomitant with increasing Treg polarization. Moreover, in a clinical setting, MSC-Exo have been tested in the treatment of resistant grade IV acute GVHD patient, which experienced improvement in symptoms for 5 months. The anti-inflammatory molecules interleukin-10, transforming growth factor-β1 and human leukocyte antigen-G contained in the exosome preparations are believed to contribute to the immunosuppressive effect of MSC-Exo^23^. In contrast, exosomes released by MSCs derived from the islets of Langerhans of nonobese diabetic (NOD) mice showed highly immunostimulatory properties to trigger autoimmune response^35^. These conflicting results might be due to the different source of MSCs and different microenvironment.

The mechanisms by which MSC-Exo suppress immunity remains unclear. It was found that although human ESC-derived MSC-Exo fail to suppress conA-stimulated mouse splenocytes effectively, they activate MYD88-dependent signaling via Toll-like receptor (TLR) 4 ligands in mouse monocytes to induce a M2-like macrophage phenotype, which in turn will polarize activated CD4^+^ T cells to CD4^+^CD25^+^FoxP3^+^ regulatory T cells^22^. However, in a separate paper, *in vitro* experiments demonstrated that human adipose MSC-Exo can exert an inhibitory effect on the proliferation, differentiation and activation of stimulated T cells^36^. In our study, no inhibitory effect of human MSC-Exo on the proliferation of either IRBP or ConA activated rat T cells was found, at any concentration applied. We also performed T cell apoptosis assay on sections of the eyes on 21 day after immunization, and no exosome-mediated effect of T cell apoptosis was found. Our results indicate that MSC-Exo do not rely on T cell inhibition and inducing apoptosis to suppress the inflammation in an autoimmune response.

To further explore how MSC-Exo inhibit ocular inflammation, we designed a chemotaxis assay to investigate the influence of MSC-Exo on inflammatory cell migration in response to chemokines. We found that MSC-Exo could effectively inhibit the migration of inflammatory cells, including CD4^+^T cells, neutrophils, NK cells, macrophages cells and significantly reduce the percentage of CD4^+^IFN-γ^+^ and CD4^+^IL-17^+^ cells in the retina. These findings suggest that periocular injection of MSC-Exo exert a beneficial effect at least partly by inhibiting leukocyte migration. Although there is a trend that Tregs decreased after exosome therapy, the reduction is of no significance, indicating that MSC-Exo therapy would not damage the beneficial role of Tregs on the regression of the disease. This decreasing trend of Tregs might result from the reduction of total peripheral inflammatory leukocytes infiltrated into the eye. It was also shown recently that micro RNA encapsulated in human MSC-Exo can inhibit the activation of mouse macrophages by suppressing TLR and NF-κB signaling, in particular MYD88-dependent pathways, and the production of inflammatory mediators central to inflammation^21^. Intravenous administration of MSC-Exo post-silica exposure significantly reduced monocyte infiltration in the lung and secretion of inflammatory mediators^21^. Given that exosomes are regarded as complex “living” structures containing a multitude of proteins, lipids, miRNAs and RNAs, which likely operate collaboratively in a mutifactorial manner, further investigations based on proteome and miRNA analysis are needed to elucidate the mechanism of their anti-inflammatory and immunomodulatory effects.

Periocular injection for therapeutic purposes is relatively safe compared with intravitreal injection, and can avoid the dilution effect of systemic delivery. Having a similar structure to synthetic lipid drug delivery vesicles presently in use, the nanoscale exosomes also have potential for penetrating through tissues and biological barriers to deliver factors to the target cells directly^6^, and therefore can be locally administered. It has been shown that after periocular injection, retinal astroglial cell-derived exosomes moved rapidly from the sub-Tenon’s space into the choroid and retina^37^.

Exosomes are naturally secreted and widely distributed in body fluids, thereby will be well tolerated by the body^38^. They can be safely stored, and provide cell free therapeutic applications avoiding the risk of immunological rejection, malignant transformation, and obstruction of small vessels associated with cell therapy^7^. The bi-lipid membrane of exosomes can maintain the encapsulated proteins and miRNAs under stable conditions to exert lasting effect^39^, and the exosomes can also be reprogrammed to be carriers for therapeutic agents^40^. Therefore, more efficient MSC-Exo based products could be developed in the future by editing MSCs or MSC-Exo to avoid the need of frequent extraocular injections.

In conclusion, we have demonstrated that exosomes released from human umbilical cord MSCs can effectively ameliorate EAU in rats by inhibiting the migration of inflammatory cells. Hence, MSC-Exo has the potential to optimize MSC-based therapy and provide a novel biologic for clinical translation in treating uveitis. However, some challenges remain to be resolved through fundamental studies, such as understanding the underlying mechanism and improving the therapeutic efficacy of MSC-Exo.

## Methods

The study was approved by the ethical committee of Tianjin Medical University Eye Hospital (2013KY(L)-10). All methods for humans were performed in accordance with the relevant guidelines and regulations.

### Animals

Male Lewis rats (6–8 weeks old) purchased from Vital River (Beijing, China) were housed under pathogen-free conditions. All procedures involving rats were approved by the Laboratory Animal Care and Use Committee of Tianjin Medical University, and conformed to the ARVO Statement for the Use of Animals in Ophthalmic and Vision Research.

### Culture and characterization of MSCs

Approximately 20 cm of umbilical cord was collected from healthy mothers after their first-term delivery (cesarean section) and immediately transferred to the laboratory (after informed patient consent) within the optimal period of 6 h in sterile normal saline solution containing 100 U/ml penicillin and 100 μg/ml streptomycin (Invitrogen, Life Technologies, Carlsbad, CA, USA). Under sterile conditions, the umbilical cord was placed in phosphate buffered saline (PBS), rinsed twice to remove blood and then cut into smaller 2 cm length pieces. Each of the pieces was washed gently and squeezed with curved forceps to remove blood trapped within the umbilical blood vessels. Sections were cut into 1–3 mm^3^ pieces and digested with collagenase type II (Sigma-Aldrich, St. Louis, MO, USA) and 0.125% trypsin in PBS for one hour. The digestion enzymes were neutralized with 10% fetal bovine serum (FBS, Invitrogen) and the cell suspension filtered through meshes to remove undigested tissue blocks. The sample was centrifuged 3 times and suspended in complete culture medium containing low-glucose Dulbecco’s modified Eagle’s medium/Nutrient Mixture F12 (Invitrogen), supplemented with 10% FBS, 100 U/ml penicillin, and 100 μg/ml streptomycin. Cells were seeded onto tissue culture flasks and expanded at 37 °C and 5% CO_2_, and the culture medium changed every 3 days. Cells were passed to a new culture flask when cultures reached 80% confluence. Cell lines from 6 healthy donors were used in the study.

In addition, MSCs were tested by flow cytometry using specific surface markers; being negative for CD34 (eBioscience, San Diego, CA, USA) and CD45 (eBioscience), and positive for CD29 (eBioscience) and CD90 (eBioscience). Mature MSCs were defined by their capacity to differentiate into adipocytes, endothelial cells, and osteocytes when cultured under appropriate conditions *in vitro*. MSCs between passages 3 and 5 were used for subsequent experiments.

Human dermal fibroblasts (Boye Biological Company, Shanghai, China) were cultured in DMEM/high glucose medium (Invitrogen) containing 10% FBS, 100 U/ml penicillin and 100 μg/ml streptomycin at 37 °C in humidified air with 5% CO_2_.

### Isolation and characterization of exosomes

Briefly, after 2 days of incubation with complete medium, MSCs and human dermal fibroblasts were cultured for 24 hours in medium containing 10% exosome-free FBS. Exosome-free FBS was prepared by sequential centrifugation at 4 °C at 200 × *g* for 10 min, 2000 × *g* for 20 min, 10000 × *g* for 30 min and 110,000 × *g* for 7 h, followed by filtration using a 0.22 μm filter. MSC-Exo were purified from culture supernatant using a sequential centrifugation procedure similar to the above, but with the last step being 110,000 × *g* for 2 h. The pellet was then resuspended in PBS and washed twice. The exosome preparation was passed through a 0.22 μm filter and stored at −20 °C until use^12, 41, 42^.

For characterization of isolated exosomes using electronic microscopy, we fixed the pellets with 2% paraformaldehyde and loaded them on Formvar-coated grids. The samples were then negatively stained with phosphotungstic acid for 1 min and examined with a Phillips CM10 electron microscope at 72,000 × magnification (Fig. 1). Exosomes secreted by human fibroblasts (Fib-Exo) were prepared in the same way as MSC-Exo and served as a control. Markers of exosomes, CD63, CD9 and CD81, were analyzed by western blot. Total proteins from the exosome pelltets were extracted in lysis buffer and protein concentrations were measured with the Protein BCA Assay Kit (solarbio, China). Samples were boiled at 95 °C for 5 min and loaded onto sodium dodecyl sulfate polyacrylamide gel electrophoresis (5% stacking gel and 12% separating gel), then transferred to PVDF membrane. The membranes were blocked with 5% nonfat dried milk and incubated in the primary antibodies overnight at 4 °C, the primary antibodies included antibodies to CD63 (Abcam, Cambridge,UK), CD9 (Abcam, Cambridge,UK), CD81(Abcam, Cambridge,UK) and β-actin (Abcam, Cambridge,UK). The membranes were incubated with secondary antibodies for 2 h. Immuno-reactive protein bands were visualized on the X-OMAT BT Film (Carestream Healt Inc, Rochester, NY, USA).

### Induction of EAU and exosome treatment protocols

EAU was induced in Lewis rats by subcutaneous immunization with 200 μl of an emulsion containing 30 μg IRBP 1177–1191 peptide called R16, ADGSSWEGVGVVPDV; Sangon, Shanghai, China) and 500 μg *Mycobacterium tuberculosis* H37RA (2.5 g/l; Difco, Detroit, MI, USA) in incomplete Freund’s adjuvant (Sigma-Aldrich), distributed over six spots on the tail base and flank. For treatment, both eyes of the immunized ratswere injected periocularly with different doses of MSC-Exo (10, 20, 50, and 100 μg), 50 μg Fib-Exos or with an equal volume of PBS on a daily basis for 7 consecutive days starting at the disease onset, usually at day 9 post immunization.

### Clinical and histological assessment of EAU

The immunized rats were examined daily for clinical signs of uveitis by slit-lamp biomicroscope starting on day 6 post-immunization. The severity of disease was scored in a masked fashion on a scale of 0–4, according to the criteria reported by Caspi’s group^43^. In brief: 0, no disease, eye is translucent and reflects light (red reflex); 0.5, dilated blood vessels in the iris; 1, engorged blood vessels in iris and abnormal pupil contraction; 2, hazy anterior chamber and decreased red reflex; 3, moderately opaque anterior chamber, but pupil still visible and dull red reflex; 4, opaque anterior chamber, obscured pupil and red reflex absent, or proptosis. For histology, animals were killed on days 15 and 20. Eyes were collected, immersed for 1 h in 4% glutaraldehyde/PBS and transferred into 10% glutaraldehyde/PBS for at least 24 h until further processing. Fixed and dehydrated tissues were embedded in paraffin wax and 4 μm sections were stained with a standard haematoxylin and eosin method. The presence of disease was evaluated in a double blinded fashion by examining four sections at different levels in each eye.

### Electroretinography

Retinal function was evaluated by scotopic electroretinography (ERG) (IRC, Chongqing, China) on day 12, 15 and 20 after immunization. Rats were dark adapted overnight. ERG was carried out in both eyes and always in the morning. After systemic anesthesia with by intraperitoneal injection of 10% chloral hydrate, one drop of 1% tetracaine was administered for topical anesthesia and pupils were dilated with 0.1% tropicamide. Topical methylcellulose (1%) was used as the conducting medium. The corneal electrode was a golden-ring. Reference and grounding needle electrodes were placed on the forehead and near the tail subcutaneously. Ten responses to 0.01 and 3.0 cd × s/m^2^ white light flash (10 μs, 0.1 Hz) from a Ganzfeld integrating sphere were amplified and averaged. The amplitude of the b-wave was measured from the trough of the a-wave to the peak of the b-wave, and the a-wave was calculated from the baseline to the first negative deflection. Implicit times of a- and b-waves were calculated from the onset of light stimulus to the peak of each wave, respectively.

### Immunohistochemistry staining

CD68 immunostaining was used to observe macrophage infiltration in the eyes. After deparaffinization and dehydration, microwave antigen retrieval was performed for 5 min prior to peroxidase quenching with 3% H_2_O_2_ in PBS for 15 min. Subsequently, sections were preblocked with 5% bovine serum albumin for 30 min and incubated with primary antibody (anti-CD68, clone ED1, AbD Serotec, Raleigh, NC, USA; 1:100) overnight at 4 °C. A negative control was treated as above except that primary antibody was replaced with PBS. After washing in PBS, sections were incubated with biotinylated secondary antibody for 30 min, and then stained with 3, 3′ -diaminobenzidine for 2–5 min. Sections were observed by photomicroscopyin duplicate by two different pathologists without knowledge of the group data.

### Flow cytometry analysis

On day 15 after immunization, cell suspensions were prepared from eyes and cervical draining lymph nodes. For intracellular staining of interferon (IFN)-γ and interleukin (IL)-17, aliquots of 2 × 10^5^ cells were pretreated for 4 to 6 h with 50 ng/ml phorbol 12-myristate 13w-acetate, l μg/ml ionomycin and 1 μg/ml brefeldin A (Sigma-Aldrich), and then incubated with anti-CD4 Ab for 30 min at 4 °C. After fixation and overnight permeabilization, the cells were stained with antibodies against IFN-γ and IL-17 (BioLegend). To detect the Foxp3^+^T cells, cells were firstly incubated with anti-CD4 and anti-CD25 Ab, then fixed and permeabilized overnight, followed by incubation with anti-Foxp3 Ab (BioLegend). The rest of the cells were stained with Abs against CD161 for NK cells, CD68 for macrophages, and Gr-1 for granulocytes respectively. All assays were performed in triplicate. Data collection was performed on a FACS Calibur flow cytometer (BD Biosciences, San Jose, CA, USA), and analyzed using flow cytometry software (FlowJo, Ashland, OR, USA).

### Chemotaxis Assay

We examined the effect of MSC-Exo on the chemotaxis of leukocytes*in vitro*. In this experiment, the indicator cells were splenocytes prepared from immunized rat on day 12 post immunization. The cells were added to the upper well of a microchemotaxis device (5 μm pore size, 24wellTranswell; Corning-Costar), and RPMI 1640 medium containing chemokine (C-C motif) ligand 2 and 21 (10 nMmacrophage chemoattractant CCL2; 200 ng/ml lymphocyte chemoattractant CCL21; BioLegend) were added to the lower well, with or without MSC-Exo (10 ug/ml). The cells that migrated to the lower well after 4 h were collected and the number of CD4^+^, CD161^+^, CD68^+^ and Gr^+^ cells were determined by flow cytometry. All assays were performed in triplicate.

### Lymphocyte proliferation assay *in vitro*

To measure the suppressive effect of MSC-Exo on IRBP-specific T cell proliferation *in vitro*, single cell suspensions were prepared on day 12 after immunization, from lymph nodes and spleens of EAU rats, and added to nylon wool columns. Nonadherent cells were collected as T cells, while the adherent cells were removed from columns after incubation on ice, and irradiated with 30 Gy, to serve as antigen presenting cells (APCs). T cells (4 × 10^5^ cells/well) were seeded in 96 well flat bottomed microtiter plates (Corning, Corning, NY, USA) with graded doses of R16 (0, 1, 10 and 30 μg/ml), in the presence of APCs (4 × 10^4^ cells/well), in a total volume of 200 μl. MSC-Exo at doses of 0, 0.1, 0.5, 1 and 10 μg/ml were added to the co-cultures. ConA (0, 2.5, 5 and 10 μg/ml) was used to measure the nonspecific inhibition of MSC-Exo at concentrations of 1 and 10 μg/ml.

In every experimental condition, each culture was performed in triplicate, and the plates were incubated in a humidified atmosphere of 5% CO_2_ at 37 °C for 48 h in RPMI 1640 (Gibco) supplemented with 10% FBS, 2 mM glutamine, 100 U/ml penicillin, 100 μg/ml streptomycin, and 50 μM β-mercaptoethanol (ICN Biomedicals, Irvine, CA, USA), and labeled with BrdU. T cell proliferation was determined by measurement of BrdU incorporation using a cell proliferation ELISA kit (Roche, Indianapolis, IN, USA) according to the manufacturer’s instructions.

### Statistical Analysis

SAS version 9.2 software (SAS, Cary, NC, USA) was used for statistical analyses. Data are expressed as means ± SD. The EAU clinical scores were assessed by repeated measures ANOVA, using mixed models. Student’s *t*-test was used for two sets of data, and 1-way ANOVA was used for three or more sets of data. Statistical significance was set at *P *<0.05.

## Electronic supplementary material

supplementary file

## References

[1] Shi Y (2012). How Mesenchymal Stem Cells Interact with Tissue Immune Responses. Trends Immunol..

[2] Liang X, Ding Y, Zhang Y, Tse HF, Lian Q (2014). Paracrine Mechanisms of Mesenchymal Stem Cell-Based Therapy: Current Status and Perspectives. Cell Transplant..

[3] Chen X (2016). CD73 Pathway Contributes to the Immunosuppressive Ability of Mesenchymal Stem Cells in Intraocular Autoimmune Responses. Stem Cells Dev..

[4] Lavoie JR, Rosu-Myles M (2013). Uncovering the Secretes of Mesenchymal Stem Cells. Biochimie..

[5] Madrigal M, Rao KS, Riordan NH (2014). A Review of Therapeutic Effects of Mesenchymal Stem Cell Secretions and Induction of Secretory Modification by Different Culture Methods. J Transl Med..

[6] Yu B, Zhang X, Li X (2014). Exosomes Derived From Mesenchymal Stem Cells. Int J Mol Sci..

[7] Rani S, Ryan AE, Griffin MD, Ritter T (2015). Mesenchymal Stem Cell-Derived Extracellular Vesicles: Toward Cell-Free Therapeutic Applications. Mol Ther..

[8] Kourembanas S (2015). Exosomes: Vehicles of Intercellular Signaling, Biomarkers, and Vectors of Cell Therapy. Annu Rev Physiol..

[9] Lai RC, Yeo RW, Lim SK (2015). Mesenchymal Stem Cell Exosomes. Semin Cell Dev Biol..

[10] Lai RC (2010). Exosome Secreted by MSC Reduces Myocardial Ischemia/Reperfusion Injury. Stem Cell Res..

[11] van Koppen A (2012). Human Embryonic Mesenchymal Stem Cell-Derived Conditioned Medium Rescues Kidney Function in Rats with Established Chronic Kidney Disease. Plos One..

[12] Li T (2013). Exosomes Derived From Human Umbilical Cord Mesenchymal Stem Cells Alleviate Liver Fibrosis. Stem Cells Dev..

[13] Lai RC, Yeo RW, Tan KH, Lim SK (2013). Mesenchymal Stem Cell Exosome Ameliorates Reperfusion Injury through Proteomic Complementation. Regen Med..

[14] Zhu YG (2014). Human Mesenchymal Stem Cell Microvesicles for Treatment of Escherichia Coli Endotoxin-Induced Acute Lung Injury in Mice. Stem Cells..

[15] Xin H (2013). MiR-133b Promotes Neural Plasticity and Functional Recovery After Treatment of Stroke with Multipotent Mesenchymal Stromal Cells in Rats Via Transfer of Exosome-Enriched Extracellular Particles. Stem Cells..

[16] Zhang B (2015). HucMSC-Exoome Mediated-Wnt4 Signaling is Required for Cutaneous Wound Healing. Stem Cells..

[17] Nakamura Y (2015). Mesenchymal-Stem-Cell-Derived Exosomes Accelerate Skeletal Muscle Regeneration. Febs Lett..

[18] Rager TM, Olson JK, Zhou Y, Wang Y, Besner GE (2016). Exosomes Secreted From Bone Marrow-Derived Mesenchymal Stem Cells Protect the Intestines From Experimental Necrotizing Enterocolitis. J Pediatr Surg..

[19] Doeppner TR (2015). Extracellular Vesicles Improve Post-Stroke Neuroregeneration and Prevent Postischemic Immunosuppression. Stem Cells Transl Med.

[20] Teng X (2015). Mesenchymal Stem Cell-Derived Exosomes Improve the Microenvironment of Infarcted Myocardium Contributing to Angiogenesis and Anti-Inflammation. Cell Physiol Biochem..

[21] Phinney DG (2015). Mesenchymal Stem Cells Use Extracellular Vesicles to Outsource Mitophagy and Shuttle microRNAs. Nat Commun..

[22] Zhang B (2014). Mesenchymal Stem Cells Secrete Immunologically Active Exosomes. Stem Cells Dev..

[23] Kordelas L (2014). MSC-derived Exosomes: A Novel Tool to Treat Therapy-Refractory Graft-Versus-Host Disease. Leukemia..

[24] Yang P (2015). Editorial: Uveitis: Pathology, Molecular Mechanisms and Therapy. Curr Mol Med..

[25] Rosenbaum JT (2010). Future for Biological Therapy for Uveitis. Curr Opin Ophthalmol..

[26] Tempest-Roe S, Joshi L, Dick AD, Taylor SR (2013). Local Therapies for Inflammatory Eye Disease in Translation: Past, Present and Future. Bmc Ophthalmol..

[27] Zhang X (2011). Mesenchymal Stem Cells Ameliorate Experimental Autoimmune Uveoretinitis by Comprehensive Modulation of Systemic Autoimmunity. Invest Ophthalmol Vis Sci..

[28] Tasso R (2012). Mesenchymal Stem Cells Induce Functionally Active T-regulatory Lymphocytes in a Paracrine Fashion and Ameliorate Experimental Autoimmune Uveitis. Invest Ophthalmol Vis Sci..

[29] Li G (2013). The Effect of Mesenchymal Stem Cells On Dynamic Changes of T Cell Subsets in Experimental Autoimmune Uveoretinitis. Clin Exp Immunol..

[30] Kimbrel EA (2014). Mesenchymal Stem Cell Population Derived From Human Pluripotent Stem Cells Displays Potent Immunomodulatory and Therapeutic Properties. Stem Cells Dev..

[31] Zhang L (2014). Long-Term Therapeutic Effects of Mesenchymal Stem Cells Compared to Dexamethasone On Recurrent Experimental Autoimmune Uveitis of Rats. Invest Ophthalmol Vis Sci..

[32] Oh JY (2014). Intraperitoneal Infusion of Mesenchymal Stem/Stromal Cells Prevents Experimental Autoimmune Uveitis in Mice. Mediators Inflamm..

[33] Ko JH (2016). Mesenchymal Stem/Stromal Cells Precondition Lung Monocytes/Macrophages to Produce Tolerance Against Allo- and Autoimmunity in the Eye. Proc Natl Acad Sci USA.

[34] Zhao, P. T. *et al*. Therapeutic Effects of Mesenchymal Stem Cells Administered at Later Phase of Recurrent Experimental Autoimmune Uveitis. *Int J Ophthalmol*. **9**, 1381–1389 (2016). 10.18240/ijo.2016.10.03 PMC5075650 27803852

[35] Rahman MJ, Regn D, Bashratyan R, Dai YD (2014). Exosomes Released by Islet-Derived Mesenchymal Stem Cells Trigger Autoimmune Responses in NOD Mice. DIABETES..

[36] Blazquez R (2014). Immunomodulatory Potential of Human Adipose Mesenchymal Stem Cells Derived Exosomes On *in Vitro* Stimulated T Cells. Front Immunol..

[37] Hajrasouliha AR (2013). Exosomes From Retinal Astrocytes Contain Antiangiogenic Components that Inhibit Laser-Induced Choroidal Neovascularization. J BIOL CHEM..

[38] Sun L (2016). Safety Evaluation of Exosomes Derived From Human Umbilical Cord Mesenchymal Stromal Cell. CYTOTHERAPY..

[39] Lai RC, Chen TS, Lim SK (2011). Mesenchymal Stem Cell Exosome: A Novel Stem Cell-Based Therapy for Cardiovascular Disease. REGEN MED..

[40] Yu B (2015). Exosomes Secreted From GATA-4 Overexpressing Mesenchymal Stem Cells Serve as a Reservoir of Anti-Apoptotic microRNAs for Cardioprotection. INT J CARDIOL..

[41] Zhu YG (2014). Human Mesenchymal Stem Cell Microvesicles for Treatment of Escherichia Coli Endotoxin-Induced Acute Lung Injury in Mice. STEM CELLS..

[42] Thery, C., Amigorena, S., Raposo, G. & Clayton, A. Isolation and Characterization of Exosomes From Cell Culture Supernatants and Biological Fluids. Curr Protoc Cell Biol. Chapter 3, 3–22 (2006). 10.1002/0471143030.cb0322s30 18228490

[43] Caspi, R. R. Experimental Autoimmune Uveoretinitis in the Rat and Mouse. Curr Protoc Immunol. Chapter 15, 15–16 (2003). 10.1002/0471142735.im1506s53 18432901

