# Author Correction: Effects of Mesenchymal Stem Cell-Derived Exosomes on Experimental Autoimmune Uveitis

**DOI:** 10.1038/s41598-018-28151-0

**Published:** 2018-06-26

**Authors:** Lingling Bai, Hui Shao, Hongxing Wang, Zhihui Zhang, Chang Su, Lijie Dong, Bo Yu, Xiteng Chen, Xiaorong Li, Xiaomin Zhang

**Affiliations:** 1 Tianjin Medical University Eye Hospital, Eye Institute &School of Optometry and Ophthalmology, Tianjin, 300384 P.R. China; 2 Department of Ophthalmology and Visual Sciences, Kentucky Lions Eye Center, University of Louisville, Louisville, KY 40202 USA; 3 Department of Ophthalmology, Chuiyangliu Hospital, Beijing, China

Correction to: *Scientific Reports* 10.1038/s41598-017-04559-y, published online 28 June 2017

There are typographical errors in the legend of Figure 3 which should read:-

“Histological assessment of the retina in EAU. Sections of the retina were stained with hematoxylin and eosin, and evaluated for histological damage on 15 and 20 days post immunization. Representative H&E staining images of PBS (A1 and C1, 15 days; A2 and C2 20 days), MSC-exo (A3, 15 days; A4, 20 days), and FIB-exo (C3, 15 days; C4, 20 days) treated groups are shown. Results are expressed quantitatively as histopathological scores (B and D). Values are expressed as the mean ± SD of six rats (12 eyes) per group. *P < 0.05”.

