# Supplementary material for: Effects of Mesenchymal Stem Cell-Derived Exosomes on Experimental Autoimmune Uveitis
Source: Sci Rep. 2017 Jun 28;7:4323. doi: 10.1038/s41598-017-04559-y (PMC5489510; doi:10.1038/s41598-017-04559-y)
Supplement: Supplementary file 1 — supplementary file [file 41598_2017_4559_MOESM1_ESM.pdf]

Effects of Mesenchymal Stem Cell-Derived Exosomes on Experimental Autoimmune Uveitis

Lingling Bai[1], Hui Shao[2], Hongxing Wang[3], Zhihui Zhang[1], Chang Su[1], Lijie Dong[1], Bo Yu[1], Xiteng Chen[1], Xiaorong Li[1*], Xiaomin Zhang[1*]

[1]Tianjin Medical University Eye Hospital, Eye Institute &School of Optometry and Ophthalmology

[2]Department of Ophthalmology and Visual Sciences, Kentucky Lions Eye Center, University of Louisville

[3]Department of Ophthalmology, Chuiyangliu Hospital, Beijing.

Correspondence and requests for materials should be addressed to: Xiaomin Zhang (email: xiaomzh@126.com) or Xiaorong Li (email: lixiaorong@tmu.edu.cn)

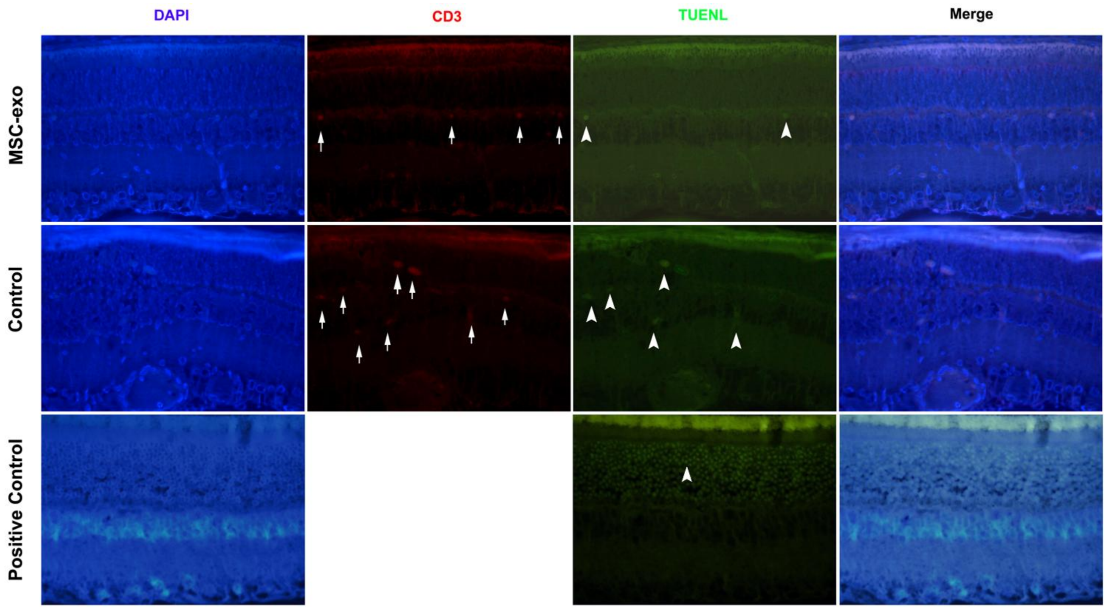

Supplementary figure: CD3 and TUENL double-fluorescence co-localization analysis of T cells apoptosis in the eyes. Blue is the cell nucleus, red indicates T cells (arrows), and green labels apoptosis (arrowheads). For positive control, fixed and permeabilized tissues were incubated with DNase Ⅰ recombinant for 10 min at room temperature to induce DNA strand breaks.
